# A Clinical Analysis of the Treatment of Chronic Coronary Artery Occlusion With Antegrade Dissection Reentry

**DOI:** 10.3389/fsurg.2021.609403

**Published:** 2021-05-31

**Authors:** Xiangjun Wu, Dan Zhang, Haitao Liu, Shuai Li, Chao Fu, Jiyuan Liu, Jiayu Cui

**Affiliations:** ^1^Department of Cardiology, Binzhou People's Hospital Affiliated to Shandong First Medical University, Binzhou, China; ^2^Department of Cardiology, Zouping People's Hospital Affiliated to Shandong First Medical University, Zouping, China; ^3^Department of Cardiology, The Second Affiliated Hospital of Nanchang University, Nanchang, China

**Keywords:** coronary chronic total occlusion, CrossBoss, antegrade dissection re-entry, percutaneous coronary intervention, major adverse cardiovascular events

## Abstract

**Objective:** This study aimed to investigate the efficacy and safety of antegrade dissection re-entry (ADR) technique in the percutaneous coronary intervention (PCI) to open chronic total occlusion (CTO) lesions.

**Methods:** The baseline, angiographic results, PCI success rate, and major adverse cardiac events (MACE) during the 12 months of follow-up were compared between 48 patients who did not use ADR in the treatment of CTO lesions (control group) and 50 patients who used ADR (treatment group).

**Results:** The control group comprised 48 patients who had 52 CTO lesions, and the treatment group comprised 50 patients who had 58 CTO lesions. The success rate of PCI in the treatment group (89.7 vs. 71.2%, *P* = 0.047) was significantly higher than in the control group, where six patients had in-stent restenosis (ISR, ISR-CTO) that were all recanalized. The mean PCI time (71 ± 25 min vs. 95 ± 33 min, *P* = 0.041), X-ray exposure time (42 ± 17 min vs. 71 ± 22 min, *P* = 0.032), contrast agent dosage (98 ± 26 ml vs. 178 ± 63 ml, *P* = 0.029), MACE incidence during the 12 months of follow-up (22.0 vs. 41.7%, *P* = 0.046) and recurrent myocardial infarction incidence (10.0 vs. 27.1%, *P* = 0.047) were significantly lower in the treatment group than in the control group. The differences were all statistically significant.

**Conclusion:** It is safe and effective to use the ADR technique in PCI for coronary artery CTO lesions. The technique shortens the operation time, reduces the radiation dose of doctors and patients and the use dose of contrast agents, and improves patients' prognoses.

## Introduction

Chronic total occlusion (CTO) of the coronary artery is defined as an occlusion lasting >3months, in which the thrombolysis in myocardial infarction (TIMI) level of forward blood flow of the occluded segment is 0. If ipsilateral collateral or collateral vessels are present, although the TIMI level of blood flow in the distal vessels is > 0, it can also be defined as CTO (1). The lesions are characterized by severe plaque load and increased hardness, which prevents the guidewire from penetrating the proximal fibrous cap of the occluded segment. They may also make the guidewire enter the intima of the vessel but not return to the true lumen of the distal segment, causing percutaneous coronary intervention (PCI) to fail. Therefore, CTO is referred to as one of the last bastions not completely conquered in PCI diagnosis and treatment (2). The antegrade dissection re-entry (ADR) specific Bridgepoint Medical System (including CrossBoss perforating catheter, Stingray^TM^ balloon, and, later, StingrayLP balloon and Stingray puncture guidewire) is specially designed for CTO lesions. In the FAST-CTO trial (3), it has been proven that the operation's success rate is significantly higher than that of the traditional interventional operation, and it has become a new strategy in the PCI treatment of CTO lesions. Clinical evidence demonstrates that successful reconstruction of blood supply in CTO can effectively improve myocardial ischemia, relieve angina pectoris (4, 5), and improve left ventricular function and clinical prognosis (6–10). At present, the application of this system in China is rare, and there is a lack of effective data in the application of CTO lesions. By analyzing the data of 50 patients with ADR, this study explores the safety and effectiveness of ADR in CTO treatment.

## Materials and Methods

### Subjects

In this randomized control trial, a total of 98 patients who had CTO lesions and were planned to receive PCI treatment at the Binzhou People's Hospital and the Second Affiliated Hospital of Nanchang University were recruited from January 2017 to December 2018. These patients were randomly divided into two groups: the treatment group (*n* = 50) and the control group (*n* = 48). For patients in the treatment group, ADR was used in PTI, while in the control group, ADR was not used during the treatment CTO lesions. The enrolled patients included those in which ADR-devices technique failed to reentry. This study was approved by the Ethics Committee of the Binzhou People's Hospital (Approval number: No.193) and performed in accordance with the Declaration of Helsinki. All patients provided written informed consent prior to the study.

### Inclusion Criteria and ADR Technical Success Criteria

#### Inclusion Criteria

(1) Complete occlusion of ≥1 blood vessel, (2) occluded vessel diameter ≥2.5 mm, (3) in-stent CTO lesions, (4) no serious diffuse lesion in the vessels far from the occluded segment, and the landing zone does not affect large branches of blood vessels, and (5) the length of the occluded segment is >20 mm (11).

#### Success Criteria for ADR Technique

The guidewire passes through the occluded segment into the true lumen of the distal segment.

### Operation Method

**Figure 1:**
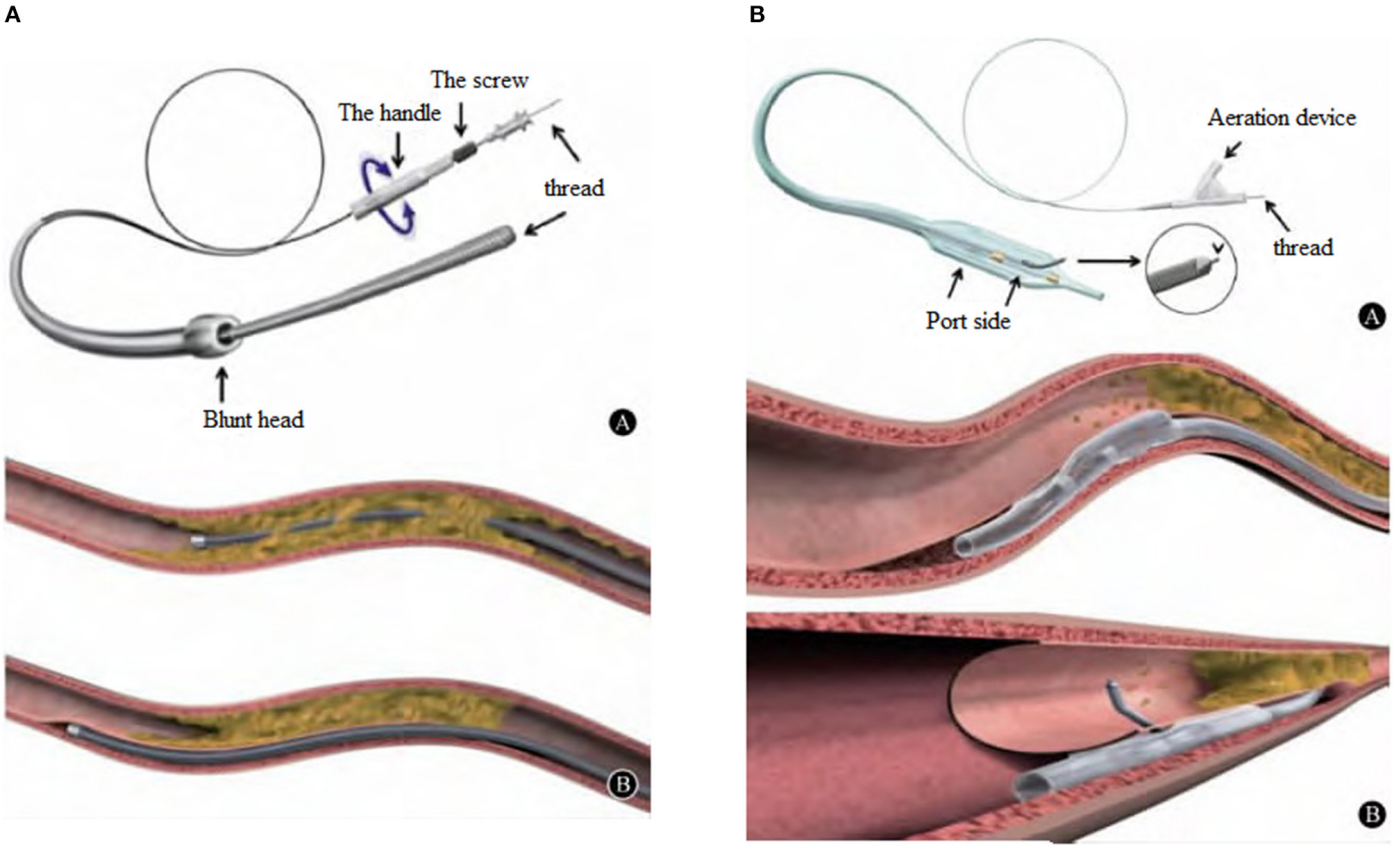
**(A)**, CrossBoss^TM^ penetrating catheter is provided by Boston Scientific Corporation in the U.S.A., with an outer diameter of 6F. Blunt tip, 3 F (1 mm) in diameter, this design allows the surgeon to use the “Fast-spin” torsion control device of the tail to quickly rotate for antegrade dissection and pass through the occlusion to reach a relatively normal blood vessel. **(B)**, CrossBoss^TM^ catheter can open the CTO lesion in two ways: One is to pass through the lumen of the blood vessel directly, and the other is to enter and open the subintimal channel.

**Figure 2:**
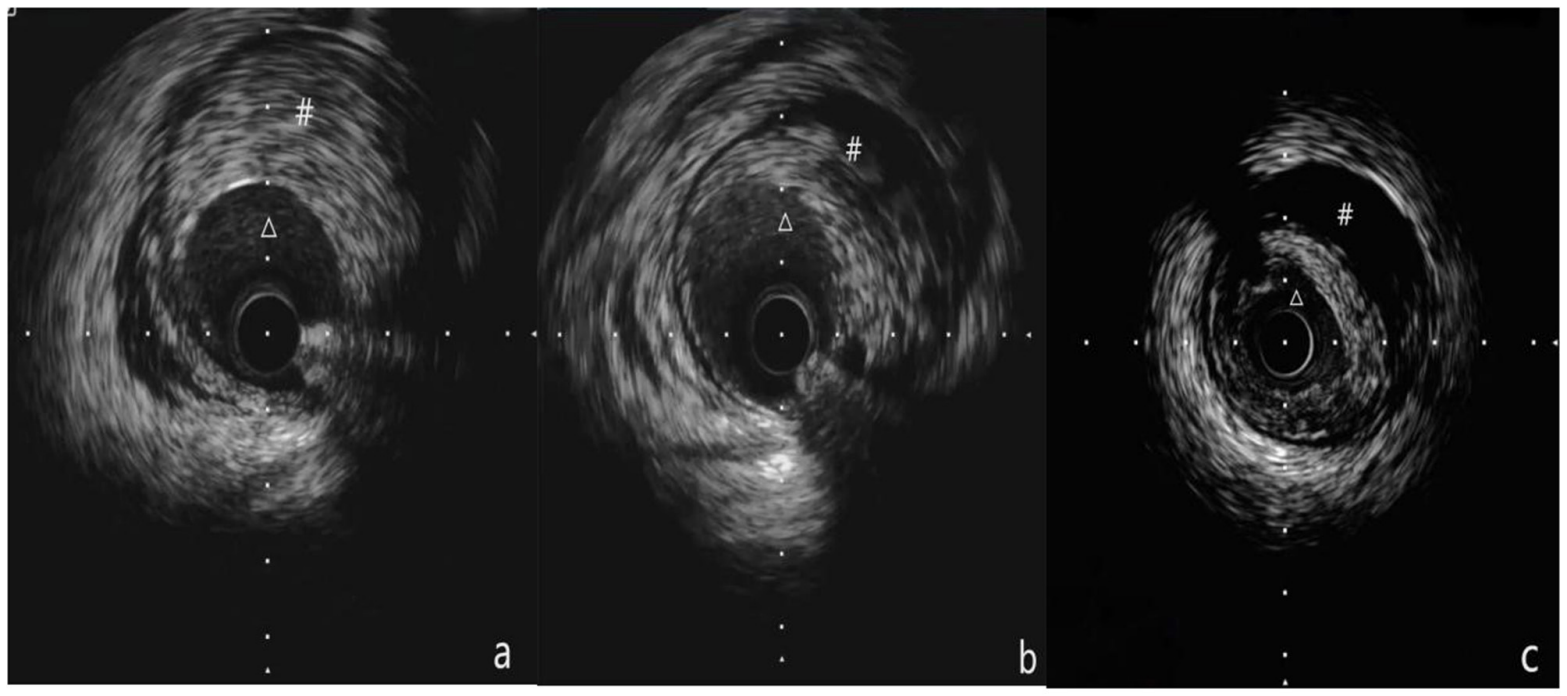
**(a-c)** Show the process of IVUS examination from the distal part to the proximal part of the blood vessel. **(a)** Shows that Δ is the true lumen of the blood vessel, # is intramural hematoma, **(b)** Shows that Δ is the true lumen of the blood vessel, # is the intramural hematoma, and **(c)** shows that Δ is the true lumen of the blood vessel, # is an intramural hematoma. **(a-c)** Show the pulling process of the IVUS catheter from far to near, showing that the guide wire travels under the intima in the middle of the blood vessel, but returns to the true lumen at the far end.

**Figure 3:**
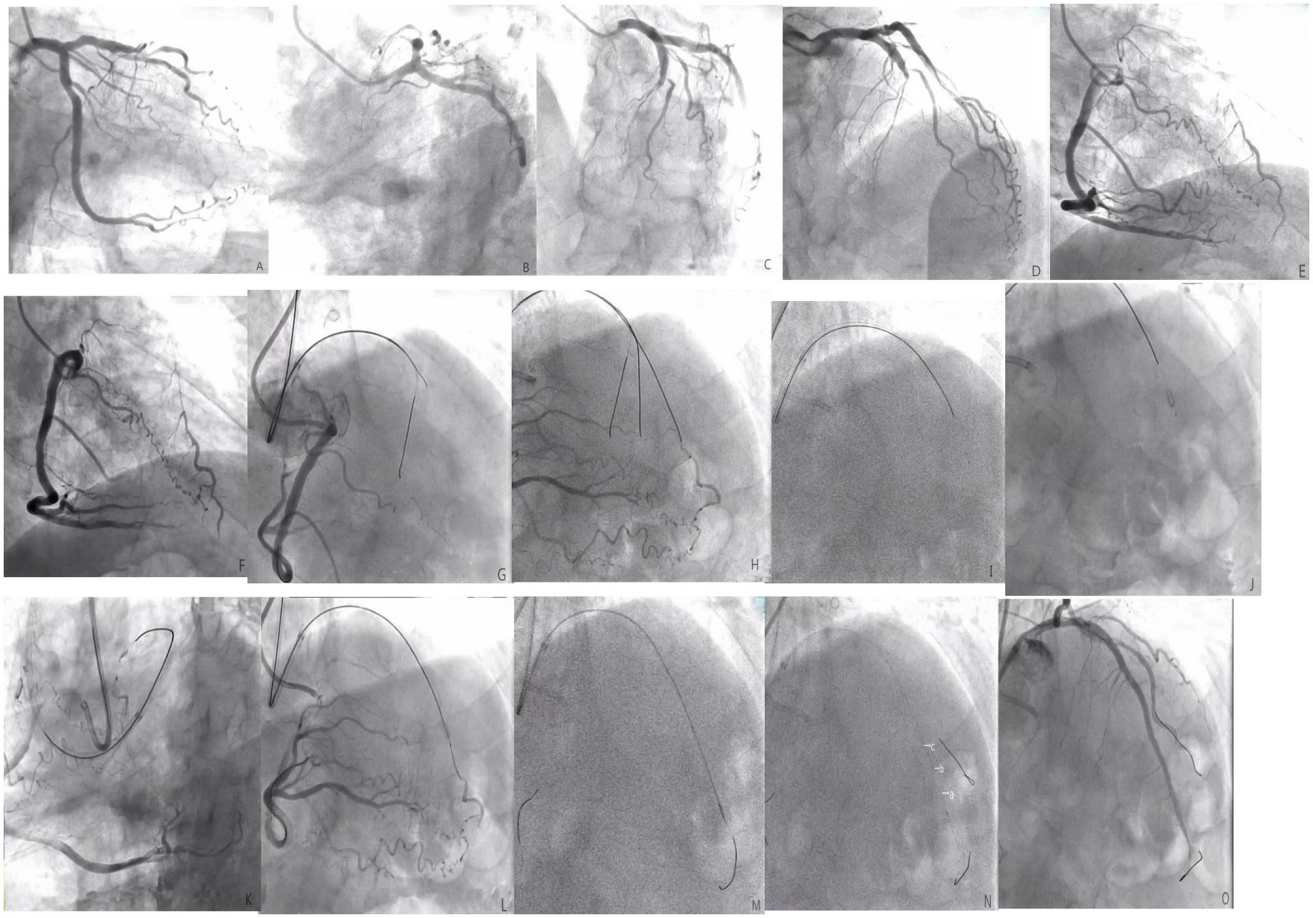
**(A–F)** The left anterior descending (LAD) is completely occluded in the near segment (the black arrow shows the stump of the LAD occlusion, and the red arrow shows the right descending coronary artery that provides the collateral circulation to the LAD); **(G)**, The guide catheter is 7F EBU3.5, the Percutaneous transluminal coronary angioplasty guidewire is SION. Corsair microcatheter is sent to the proximal end of LAD occlusion segment under the guidance of SION, XT-R finger guidewire is replaced, which does not pass through LAD occlusion lesion (shown by black arrow); **(H)**, upgrade the guidewire several times and finally allow the Conquest Pro guidewire to pass through the LAD to occlude the lesion, but the retrograde angiography shows that the guidewire is not in the true lumen of the distal vessel, but within the vessel structure (shown by the black arrow); **(I)**, start ADR technique, CrossBoss^TM^ penetrates the catheter to the proximal fiber cap of the LAD occlusion segment, and use the “fast-spin” technique to advance the CrossBoss^TM^ catheter to the LAD distal segment. The contralateral angiography prompts the CrossBoss^TM^ catheter to pass through the occluded LAD segment, but located under the endometrium of the distal segment of the occlusion segment (shown by the black arrow); **(J)**, replace the Miracle12 guidewire, withdraw the CrossBoss^TM^ catheter, enter and fill the Stingray^TM^ balloon, and display a “dual-rail sign,” indicating that the puncture position is not ideal (black arrow); **(K–M)**, change the position of the projection and display the “monorail sign,” indicating that the puncture position is ideal. The Stingray guidewire puncture is successful. The contralateral angiography shows that the guidewire is located in the true lumen of the distal vessel (shown by the black arrow), replace the SION guidewire; **(N)**, perform IVUS check [figures **(A–C)** showed that Δ was the true lumen of the blood vessel and # was intramural hematoma]; **(O)**, LAD distal to the proximal section have placed three drug-eluting stents after the final angiography results.

The CrossBoss catheter was pushed to the proximal end of CTO lesions with a guidewire, and the head propped against the proximal fibrous cap. The “Fast-spin” twist control device at its tail was used for rapid rotation to separate the antegrade dissection, allowing the guidewire to pass through the occluded segment to the relatively normal blood vessel. The CrossBoss catheter was then drawn out, and the guidewire retained. The Stingray^TM^ balloon was sucked three times using a 2-ml screw syringe, expelling air from the side hole. Pure contrast reagent was inhaled into the Stingray^TM^ balloon under negative pressure, and the Stingray^TM^ balloon was delivered along the guidewire. The guidewire was withdrawn, and the balloon was filled with 6 atm (1 atm = 101.325 kpa) of contrast agent, allowing the Stingray^TM^ balloon to be filled under the intima and encircle the blood vessels. There are two 180° opposite outlets on the Stingray^TM^ balloon, one of which points to the true lumen of blood vessels when being filled. At this time, fluoroscopy was performed in different positions. When the Stingray^TM^ balloon presented with the “monorail sign,” the Stingray puncture guidewire (or Conquest Pro guidewire) was used to penetrate the vascular intima. When the contralateral angiography confirmed that the guidewire was located in the true lumen of the blood vessel in at least two positions, the Stingray^TM^ balloon was sucked and deflated, trapping was removed, the microcatheter expanded, and the working guidewire was switched to the distal true lumen to complete the subsequent PCI operations (see Figures 1–3).

### Follow-Up

All patients were followed up by an outpatient appointment or telephone call. The follow-up endpoints were MACE, including death, recurrent angina pectoris, myocardial infarction, target vessel reconstruction, and in-stent thrombosis.

### Statistical Analysis

Data were statistically analyzed using software SPSS19.0. Normally distributed measurement data were expressed as mean ± standard deviation (x ± SD) and, then, compared between the two groups using a *t*-test or *U*-test. Non-normally distributed measurement data were expressed as the median [25% percentile, 75% percentile, M ([Q25, Q75])], and compared between the two groups using non-parametric rank-sum tests. Count data were expressed as percentages (%) and compared using a Chi-square test. *P* < 0.05 was considered statistically significant.

## Results

### Comparison of Baseline Data Between the Two Groups

The differences in age, gender, risk factors, hypertension, hyperlipidemia, and disease history between the two groups were not statistically significant (*P* > 0.05 for all; Table 1).

**Table 1:** Comparison of baseline characteristics between the two groups.

**Characteristics**	**Control group (*n* = 48)**	**Treatment group (*n* = 50)**	***P-*value**
Age (mean ± SD, years)	54.9 ± 8.93	56.7 ± 9.98	NS
Male [case (%)]	32 (66.7)	35 (70.0)	NS
Smoking history [case (%)]	23 (47.9)	25 (50.0)	NS
hypertension [case (%)]	30 (62.5)	32 (64.0)	NS
hyperlipidemia [case (%)]	20 (41.7)	21 (42.0)	NS
Stroke [case (%)]	2 (4.2)	2 (4.0)	NS
Family history of premature coronary heart disease [case (%)]	2 (4.2)	3 (6.0)	NS
History of myocardial infarction [case(%)]	13 (27.1)	14 (28.0)	NS
History of angina [case (%)]	28 (58.3)	30 (60.0)	NS
History of heart failure [case (%)]	3 (6.25)	4 (8.0)	NS
History of PCI [case (%)]	7 (14.6)	8 (16.0)	NS
PCI failed to treat CTO lesions [case (%)]	4 (8.33)	5 (10.0)	NS
History of CABG [case (%)]	1 (2.1)	1 (2.0)	NS

*PCI, percutaneous coronary intervention; CTO, Chronic total occlusion; CABG, Coronary artery bypass grafting*.

### Comparison of CTO Pathological Features and PCI Success Rate Between the Two Groups

The differences in the characteristics of diseased vessels of CTO between the two groups were not statistically significant (*P* > 0.05). The success rate of PCI in the treatment group (89.7 vs. 71.2%, *P* = 0.047) was significantly higher than that in the control group. The difference was statistically significant. The mean PCI time (71 ± 25 vs. 95 ± 33 min, P = 0.041), X-ray exposure time (42 ± 17 vs. 71 ± 22 min, *P* = 0.032), and contrast agent dosage (98 ± 26 ml vs. 178 ± 63 ml, *P* = 0.029) were significantly lower in the treatment group than in the control group (Table 2).

**Table 2:** Comparison of CTO pathological features and PCI success rate between the two groups.

**MACE results**	**Control group (*****n*** **=** **48)**		**Treatment group (*****n*** **=** **50)**		***x*^**2**^/t value**	***P-*value**
	**Incidence rate (52 places)**	**Success (37 places)**	**Incidence rate (58 places)**	**Success (52 places)**		
**The CTO target blood vessels [place (%)]**						
Left anterior descending branch	22 (42.3)	15 (68.2)	24 (41.2)	20 (83.3)	3.987	0.047
Cyclotron branch	7 (13.5)	5 (71.4)	8 (13.8)	8 (100)	5.175	0.031
Right coronary artery	23 (44.2)	17 (73.9)	26 (44.8)	24 (92.3)	4.981	0.039
**Vascular characteristics of CTO lesions [place (%)]**						
Moderate or severe tortuosity	3 (5.8)	1 (33.3)	4 (6.9)	1 (25.0)	0.637	NS
Moderate or severe calcification	3 (5.8)	1 (33.3)	2 (3.4)	1 (50.0)	2.317	NS
**Collateral circulation [place (%)]**						
Contralateral collateral circulation supplies blood	10 (19.2)	5 (50.0)	11 (19.0)	9 (81.8)	6.117	0.011
Ipsilateral collateral circulation supplies blood	5 (9.6)	2 (40.0)	9 (15.5)	8 (88.9)	6.846	0.009
Both the ipsilateral and contralateral sides have collateral blood supply	8 (15.4)	5 (62.5)	10 (17.2)	10 (100)	6.481	0.007
**Anatomical morphology of occluded end [place (%)]**						
Tapered end	12 (23.1)	8 (66.7)	14 (24.1)	13 (92.9)	6.347	0.006
Keep occlusion	11 (21.2)	8 (72.7)	15 (25.9)	13 (86.7)	3.168	0.049
Other shapes	29 (55.8)	21 (72.4)	29 (50.0)	26 (89.7)	3.281	0.049
**The mean PCI time (mean** **±** **SD, min)**	107 ± 38	95 ± 33	85 ± 31	71 ± 25	1.997	0.041
**X-ray exposure time (mean** **±** **SD, min)**	77 ± 25	71 ± 22	61 ± 19	42 ± 17	2.231	0.032
**Contrast agent dosage (mean** **±** **SD, ml)**	223 ± 69	178 ± 63	117 ± 31	98 ± 26	4.127	0.029
**In-stent CTO lesions [place (%)]**	4 (7.7)	1 (25.0)	6 (10.3)	6 (100)	6.014	0.006
**The success rate of PCI [place (%)]**	37 (71.2)		52 (89.7)		3.894	0.047

*NS, P > 0.05; Operative time was defined as the time between the catheter reaching the coronary artery and leaving the coronary artery at the end of the operation*.

The failure in the treatment of six lesions are summarized as follows: (1) The CrossBoss catheter failed to pass through the diseased segment of CTO; (2) the Stingray^TM^ balloon failed to pass through the diseased segment of CTO; and (3) Stingray guidewire could not penetrate the true lumen (the fiber cap of the landing area was tough, and the Stingray^TM^ balloon could not point to the true lumen, resulting in the formation of a large hematoma). Other possible reasons for failure apart of the devices related ones included repeated attempts to open CTO, serious calcification of CTO entrance, and no obvious collateral circulation.

The reasons of CTO failure in the control group included tortuosity, calcification, occlusion, and insufficient collateral circulation in the occluded segment.

### Comparison of MACE Results in 12 Months of Follow-Up Between the Two Groups

The differences in the incidence of MACE during hospitalization and at 30 days and 6 months after discharge, between the two groups, were not statistically significant (*P* > 0.05). The incidence of MACE (22.0 vs. 41.7%, *P* = 0.046) and recurrent myocardial infarction (10.0 vs. 27.1%, *P* = 0.047) in 12 months of follow-up in the treatment group were significantly lower than those in the control group. The differences were statistically significant (Table 3).

**Table 3:** Comparison of MACE results between the two groups during 12-month follow-up [cases (%)].

**MACE results**	**Control group (*n* = 48)**	**Treatment group (*n* = 50)**	***P-*value**
Hospitalization MACE	2 (4.17)	2 (4.0)	NS
Perioperative PCI - related myocardial infarction	1 (2.08)	1 (2.0)	NS
Blood flow rebuilt again	1 (2.08)	1 (2.0)	NS
Cardiac death	0 (0)	0 (0)	-
30d of follow-up MACE	4 (8.3)	2 (4.0)	NS
Reoccurrence of myocardial infarction	2 (4.17)	2 (4.0)	NS
Blood flow rebuilt again	2 (4.17)	1 (2.0)	NS
Cardiac death	0 (0)	0 (0)	-
6 months of follow-up MACE	8 (16.7)	7 (14.0)	NS
Reoccurrence of myocardial infarction	3 (6.25)	3 (6.0)	NS
Blood flow rebuilt again	3 (6.25)	3 (6.0)	NS
Cardiac death	2 (4.17)	1 (2.0)	NS
12 months of follow-up MACE	20 (41.7)	11 (22.0)	0.046
Reoccurrence of myocardial infarction	13 (27.1)	5 (10.0)	0.047
Blood flow rebuilt again	4 (8.3)	4 (8.0)	NS
Cardiac death	3 (6.25)	2 (4.0)	NS

*MACE, Major adverse cardiovascular events*.

## Discussion

At present, there are three categories of recanalization techniques for CTO lesions: antegrade guidewire upgrade technique, retrograde guidewire upgrade technique, and ADR technique. The retrograde guidewire upgrade technique includes retrograde guidewire passing technique, antegrade and retrograde guidewire butt joint technique, controlled antegrade and retrograde subendocardial tracking (CART) technique, and retrograde CART technique. In the United States, 35–40% of patients with CTO lesions are treated with antegrade guidewire upgrade technique. Approximately 20% of patients are treated with retrograde guidewire upgrade technique, and 30% of patients received ADR treatment (12). The application of new techniques pushes CTO PCI to a higher level (13). However, the use of ADR technique in China is limited, so we need to accumulate clinical experience in more interventional practices.

ADR technique (14) addresses the issue that during the antegrade interventional treatment for the CTO lesions, the guidewire cannot enter the distal vascular true lumen through the occluded lesions. BridgePoint Medical System, a specialized CTO system, is used to control the guidewire to re-enter the distal vascular true lumen through the subintima of the vessels. In some cases, the guidewire can be directed to enter the true lumen from the false lumen under the guidance of intravascular ultrasound (IVUS) (15). ADR technique is an improvement of early subintimal tracking and re-entry (STAR) technique. STAR technique refers to the use of guidewire to tear a gap at the intima of blood vessels (including Knuckle technique) and control the guidewire to re-penetrate from subintima and enter the true lumen of blood vessels. The disadvantages of STAR technique are the poor controllability of the guidewire and that the subintimal hematoma it induces can damage the distal branches of blood vessels (16), resulting in a failure of the guidewire to return to the true lumen. However, ADR should be avoided if the occluded distal vessels are tortuous, calcified, and have large branches. Because it can reduce the forward pushing force and penetration force of the CrossBoss catheter, the subintimal pseudolumen caused by the CrossBoss catheter compresses the distal vascular true lumen, meaning the guidewire cannot return to the true lumen.

A meta-analysis of using ADR technique to recanalize CTO lesions revealed that the success rate of recanalization of CTO was 77%, and the risk of surgical complications did not increase (12). A FAST-CTO study revealed that (3), the success rate of ADR was significantly higher than that of standard PCI (77 vs. 59%, *P* < 0.001), and the incidence of 30-day MACE was similar (4.8 vs. 6.9%, P = 0.40). Mogabgab (17) conducted a long-term follow-up of 170 patients with CTO lesions after ADR. The average follow-up duration was 1.81 years. The result revealed that the rate of target vessel reconstruction (40.9 vs. 29.6%, *P* = 0.13) and the incidence of MACE (40.3% vs. 35.2%, *P* = 0.42) were not significantly different from those of standard PCI. This confirms that the ADR technique has good safety results in CTO treatment.

In the present study, ADR was used in 58 CTO lesions of 50 patients. The success rate of PCI in the treatment group (89.7 vs. 71.2%, *P* = 0.047) was significantly higher than that in the control group, which greatly improved the success rate of antegrade recanalization of CTO lesions. In addition, PCI duration, X-ray exposure time, and contrast agent dosage were reduced. The risk of contrast-induced nephropathy was also reduced. The results of the present study suggest that ADR can improve the prognosis of patients 12 months after the operation and reduce the incidence of MACE, which has some advantages compared with the traditional operation method.

The external diameter of the head end of the CrossBoss catheter is 1 mm, which is a non-invasive blunt circular structure. This head end design allows it to avoid moving between the stent steel beam and the vascular wall. Wilson (18) used ADR to treat 30 cases of CTO due to in-stent restenosis (ISR-CTO), and the success rate was 90%. In the present study, all six cases of ISR-CTO were recanalized, suggesting that it has a unique advantage in the treatment of ISR-CTO lesions.

At present, there is a lack of experience in the large-scale use of the ADR technique in China, and some major cardiac intervention centers need to learn from the technical methods of European and American countries. ADR technical processes in European and American countries are as follows (19): (1) Basic bilateral coronary angiography; (2) using a CrossBoss catheter or Knuckle-boss technique to reach the subintima of the vascular true lumen through the diseased segment of CTO; (3) using Miracle guidewire to guide the Stingray^TM^ balloon; (4) seeking the tangent position that makes the Stingray^TM^ balloon present with “monorail sign,” to determine the direction of the true lumen of blood vessels by retrograde coronary angiography, using Stingray guidewire to puncture into the true lumen, and the Pilot200 exchanged to guide the wire into the true lumen at a distance; and (5) exchanging the Stingray guidewire for a working guidewire through Corsair microcatheter, thereby completing the subsequent routine PCI treatment. The disadvantages of this process are as follows: (1) The CrossBoss catheter or Knuckle-boss technique may cause a large subintimal hematoma at the distal end of some CTO lesions. As a result, the Stingray^TM^ balloon cannot point to the vascular true lumen. Stingray guidewire cannot penetrate the true lumen, so when the antegrade guidewire was too close to the true lumen, we tried to use Corsair microcatheter. The external diameter of the head end of a Corsair microcatheter is 0.87 mm, and the external diameter of the body is 0.93 mm. This is equivalent to the external diameter of a CrossBoss catheter (1 mm) and has the advantage of tapering the head end, so it will not cause a significant subintimal hematoma; and (2) there is a probe with a length of 0.18 mm at the head end of Stingray guidewire. The pre-molding angle of the head end is only 28°, making it difficult for the guidewire to return to the true lumen in some cases. In this study, the Conquest Pro guidewire was chosen as the puncture guidewire, and success was achieved. We modified the surgical procedures introduced by the teams from Europe and the United States and achieved success.

The measures that could be used to prevent failure in the treatment are as follows: (1) Subintimal small balloon expansion or Knuckle-wire technique can be used; (2) Corsair microcatheter can be used if the Stingray^TM^ balloon channel is well-prepared, the tissue of CTO lesions is soft, and the patients with distortion and calcification of blood vessels need a recanalization of the channel with a CrossBoss catheter. The Stingray^TM^ balloon being parallel to the vascular cavity is the most important factor for the success of the puncture. If there are severe, unusual plaques in the landing area, or small lumen or Stingray^TM^ balloons located in vascular angulation, it is necessary to replace the flat and thicker segment of the lumen for puncture. If the true lumen of the blood vessel is not clear, we can only slide the Stingray^TM^ balloon back and forth for trial-and-error puncture; and (3) if the landing area fiber cap is tough and the Stingray guidewire cannot penetrate into the true lumen, we can try to stick, swap or replace Conquest Pro12 and Conquest Pro8-20 guidewire, so it can slide forward and puncture at the weak point in front. The large hematoma causes the Stingray^TM^ balloon to float in it, unable to provide stable support. In addition, the lumen near the landing area is pressed, resulting in a puncture failure. In this case, a Corsair microcatheter can be introduced for aspiration of the hematoma, or the Conquest Pro guidewire can also be used to slide forward for puncture.

Our experience for ADR is summarized as follows: (1) It is important to prevent the formation of hematoma and control the expansion of hematoma in ADR; (2) avoiding too many, or forcing an antegrade coronary angiography, if possible, means that high selective angiography or contralateral coronary angiography can be performed via ipsilateral collateral vessels; (3) avoiding frequent and large angle rotating guidewire; and (4) in cases of parallel guidewire technique, once it is found that the hematoma extends to the distal end, the strategy conversion should be considered in time.

There are some limitations to the current study. First, we did not perform power analysis to determine sample size. Second, the number of patients recruited to this study was relatively small. Further investigations with larger samples size and a multi-center design are needed to verify the results from the current study.

In conclusion, our study shows that ADR shortens the operation time, reduces the radiation dose of doctors and patients and the use dose of contrast agents, and improves patients' prognoses. Therefore, the ADR technique may be a safe and effective technique in PCI treatment for coronary artery CTO lesions.

## Data Availability Statement

The raw data supporting the conclusions of this article will be made available by the authors, without undue reservation.

## Ethics Statement

The studies involving human participants were reviewed and approved by Ethics Committee of Binzhou People's Hospital. The patients/participants provided their written informed consent to participate in this study.

## Author Contributions

XW and DZ: conception and design of the research. XW and CF: acquisition of data. XW and HL: analysis and interpretation of the data. DZ and SL: statistical analysis. XW and JL: writing of the manuscript. JC and CF: critical revision of the manuscript for intellectual content. All authors contributed to the article and approved the submitted version.

## Conflict of Interest

The authors declare that the research was conducted in the absence of any commercial or financial relationships that could be construed as a potential conflict of interest.
